# An octopamine-specific GRAB sensor reveals a monoamine relay circuitry that boosts aversive learning

**DOI:** 10.1093/nsr/nwae112

**Published:** 2024-03-26

**Authors:** Mingyue Lv, Ruyi Cai, Renzimo Zhang, Xiju Xia, Xuelin Li, Yipan Wang, Huan Wang, Jianzhi Zeng, Yifei Xue, Lanqun Mao, Yulong Li

**Affiliations:** State Key Laboratory of Membrane Biology, School of Life Sciences, Peking University, Beijing 100871, China; State Key Laboratory of Brain and Cognitive Science, Institute of Biophysics, Chinese Academy of Sciences, Beijing 100101, China; PKU-IDG/McGovern Institute for Brain Research, Beijing 100871, China; State Key Laboratory of Membrane Biology, School of Life Sciences, Peking University, Beijing 100871, China; PKU-IDG/McGovern Institute for Brain Research, Beijing 100871, China; State Key Laboratory of Membrane Biology, School of Life Sciences, Peking University, Beijing 100871, China; PKU-IDG/McGovern Institute for Brain Research, Beijing 100871, China; Yuanpei College, Peking University, Beijing 100871, China; Peking-Tsinghua Center for Life Sciences, Academy for Advanced Interdisciplinary Studies, Peking University, Beijing 100871, China; State Key Laboratory of Membrane Biology, School of Life Sciences, Peking University, Beijing 100871, China; PKU-IDG/McGovern Institute for Brain Research, Beijing 100871, China; Peking University–Tsinghua University–National Institute of Biological Sciences Joint Graduate Program, Academy for Advanced Interdisciplinary Studies, Peking University, Beijing 100871, China; State Key Laboratory of Membrane Biology, School of Life Sciences, Peking University, Beijing 100871, China; PKU-IDG/McGovern Institute for Brain Research, Beijing 100871, China; State Key Laboratory of Membrane Biology, School of Life Sciences, Peking University, Beijing 100871, China; PKU-IDG/McGovern Institute for Brain Research, Beijing 100871, China; State Key Laboratory of Membrane Biology, School of Life Sciences, Peking University, Beijing 100871, China; PKU-IDG/McGovern Institute for Brain Research, Beijing 100871, China; State Key Laboratory of Membrane Biology, School of Life Sciences, Peking University, Beijing 100871, China; PKU-IDG/McGovern Institute for Brain Research, Beijing 100871, China; Institute of Molecular Physiology, Shenzhen Bay Laboratory, Shenzhen 518107, China; College of Chemistry, Beijing Normal University, Beijing 100875, China; College of Chemistry, Beijing Normal University, Beijing 100875, China; State Key Laboratory of Membrane Biology, School of Life Sciences, Peking University, Beijing 100871, China; PKU-IDG/McGovern Institute for Brain Research, Beijing 100871, China; Yuanpei College, Peking University, Beijing 100871, China; Peking-Tsinghua Center for Life Sciences, Academy for Advanced Interdisciplinary Studies, Peking University, Beijing 100871, China; Peking University–Tsinghua University–National Institute of Biological Sciences Joint Graduate Program, Academy for Advanced Interdisciplinary Studies, Peking University, Beijing 100871, China; Institute of Molecular Physiology, Shenzhen Bay Laboratory, Shenzhen 518107, China; Chinese Institute for Brain Research, Beijing 102206, China

**Keywords:** octopamine, dopamine, GRAB sensor, learning and memory

## Abstract

Octopamine (OA), analogous to norepinephrine in vertebrates, is an essential monoamine neurotransmitter in invertebrates that plays a significant role in various biological functions, including olfactory associative learning. However, the spatial and temporal dynamics of OA *in vivo* remain poorly understood due to limitations associated with the currently available methods used to detect it. To overcome these limitations, we developed a genetically encoded GPCR  activation-based (GRAB) OA sensor called GRAB_OA1.0_. This sensor is highly selective for OA and exhibits a robust and rapid increase in fluorescence in response to extracellular OA. Using GRAB_OA1.0_, we monitored OA release in the *Drosophila* mushroom body (MB), the fly's learning center, and found that OA is released in response to both odor and shock stimuli in an aversive learning model. This OA release requires acetylcholine (ACh) released from Kenyon cells, signaling via nicotinic ACh receptors. Finally, we discovered that OA amplifies aversive learning behavior by augmenting dopamine-mediated punishment signals via Octβ1R in dopaminergic neurons, leading to alterations in synaptic plasticity within the MB. Thus, our new GRAB_OA1.0_ sensor can be used to monitor OA release in real time under physiological conditions, providing valuable insights into the cellular and circuit mechanisms that underlie OA signaling.

## INTRODUCTION

Octopamine (OA) is an essential monoamine neurotransmitter in invertebrates, analogous to norepinephrine (NE) in vertebrates [1,2]. In vertebrates, OA is classified as a trace amine and is thought to be associated with emotional responses [3–5]. In invertebrates, OA plays a role in various physiological processes, including the sleep–wake cycle, flight, ovulation, aggression and associative learning [6–27].

In *Drosophila melanogaster*, OA has been implicated in regulating both learning and memory, particularly in the formation of short-term associative memories of an odor-conditioned stimulus (CS) paired with either an appetitive sugar reward or an aversive electrical body shock as the unconditioned stimulus (US). Moreover, studies have shown that mutants lacking tyramine β hydroxylase (TβH), the rate-limiting enzyme for OA biosynthesis, have an impaired ability to acquire appetitive memory [19]. Furthermore, stimulation of octopaminergic neurons (OANs) can replace sugar presentation during conditioning and lead to the formation of short-term appetitive memory [20,21]. However, studies regarding aversive conditioning have yielded conflicting results. For example, some studies found normal performance in TβH mutants [19,28], while other studies found impaired performance when compared with wild-type (WT) flies [29].

In the *Drosophila* brain, the mushroom body (MB) is the main center for olfactory learning [30–33] and consists primarily of Kenyon cells (KCs), with their dendrites residing in the calyx and their axon bundles projecting through the peduncle to form the α/β lobe, α’/β’ lobe and γ lobe [34–36]. Studies have shown that OA signaling via the β-adrenergic-like OA receptor Octβ1R is required for aversive memory formation in the MB [25]. In addition to its role in short-term memory, OA released from the anterior paired lateral (APL) neurons has been shown to modulate intermediate-term aversive memory by acting on KCs via Octβ2R [23]. Together, these findings suggest that OA indeed plays a key role in aversive learning and memory in *Drosophila*. However, there are still many unresolved issues regarding the spatio-temporal dynamics of OA release and the specific role OA plays in aversive learning that warrant further investigations.

Our relatively limited understanding of how OA functions spatially and temporally during learning is primarily due to limitations in current detection methods. Traditional methods, such as microdialysis-coupled biochemical analysis [37–39], offer high specificity but low temporal resolution and complex sampling procedures, especially in invertebrates. On the other hand, electrochemical techniques such as fast-scan cyclic voltammetry (FSCV) enable rapid monitoring of endogenous OA release [40,41], but they cannot distinguish between OA and other structurally similar neurotransmitters, particularly its biological precursor tyramine (TA), which differs from OA by only one hydroxyl group and also serves as an important monoamine in invertebrates [2].

To overcome these limitations, we developed a novel G protein-coupled receptor (GPCR) activation-based (GRAB) OA sensor, utilizing the *Drosophila* Octβ2R as the sensing module and circularly-permutated enhanced green fluorescent protein (cpEGFP) as the reporter; we call this sensor GRAB_OA1.0_ (hereafter referred to as OA1.0). We found that this sensor is highly specific to OA, has sub-second kinetics and exhibits a peak increase in fluorescence of ∼660% in response to OA. Using OA1.0, we then measured spatio-temporal changes of OA in the *Drosophila* MB in response to odor and shock stimuli. Our findings reveal that the release of OA in the MB promotes the release of dopamine (DA), which increases the fly's perception of the US, thereby facilitating aversive learning.

## RESULTS

### Development and characterization of GRAB_OA1.0_

**Figure 1.**
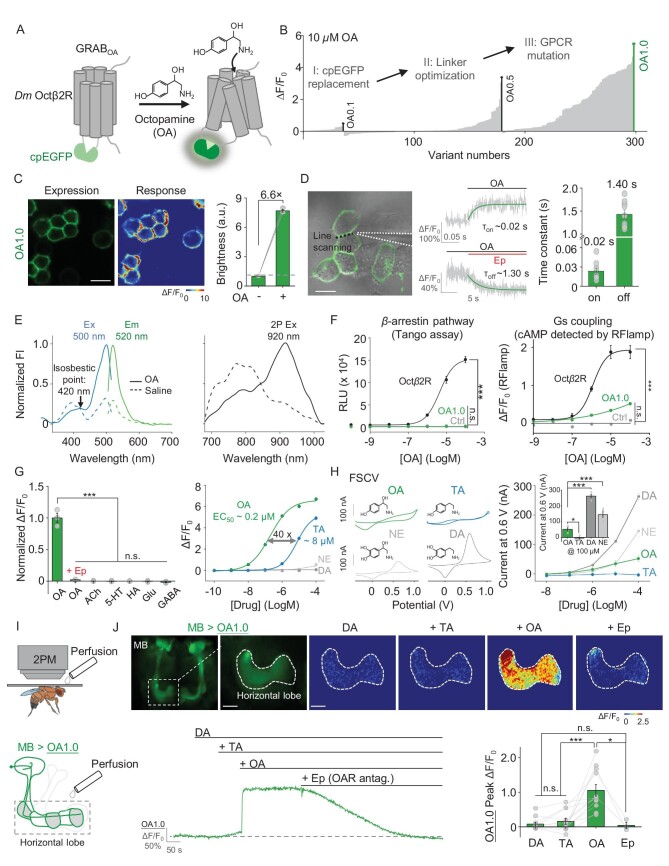
Development and characterization of the GRAB_OA1.0_ (OA1.0) sensor in HEK293T cells and living flies. (A) Schematic illustration depicting the strategy for developing the GRAB_OA_ sensor. Ligand binding activates the sensor, inducing a change in EGFP fluorescence. (B) Screening and optimization steps of GRAB_OA_ sensors and the resulting change in fluorescence (ΔF/F_0_) in response to 10 μM of OA. (C) Expression, fluorescence change in response to 100 μM of OA and summary data measured in HEK293T cells expressing OA1.0; *n* = 3 wells containing >500 cells each. (D) τ_on_ and τ_off_ were measured in OA1.0-expressing cells in response to OA and epinastine (Ep), respectively, in line-scan mode; an example image (left), representative traces (middle) and summary data (right) are shown; *n* ≥ 9 cells from three cultures; the dotted black line in the image indicates the line-scanning region. (E) One-photon (1P) excitation (ex) and emission (em) spectra (left) and two-photon (2P) excitation spectra (right) of OA1.0 were measured in the absence and presence of OA; FI, fluorescence intensity. (F) Left: The Tango assay was used to measure β-arrestin-mediated signaling in cells expressing OA1.0 or wild-type (WT) Octβ2R and treated with increasing concentrations of OA; *n* = 3 wells containing >1000 cells each. Right: The RFlamp assay was used to measure Gs coupling in cells expressing OA1.0 or Octβ2R; *n* = 3 wells containing >30 cells each. (G) Left: Normalized change in fluorescence measured in OA1.0-expressing cells in response to the indicated compounds applied at 10 μM (except Ep, which was applied at 100 μM); *n* = 3 wells containing >300 cells each. Right: Dose–response curves measured in OA1.0-expressing cells in response to OA, tyramine (TA), dopamine (DA) and norepinephrine (NE), with the corresponding EC_50_ values shown; *n* = 3 wells containing >300 cells each. ACh, acetylcholine; Glu, glutamate; GABA, γ-aminobutyric acid. (H) Left: Exemplar cyclic voltammograms for 100 μM of OA, TA, DA and NE measured using fast-scan cyclic voltammetry (FSCV); the traces were averaged from separate trials. Right: The voltammetric current responses at 0.6 V were measured in accordance with the increasing concentrations of OA, TA, DA and NE; the inset shows the summary data in response to 100 μM of OA, TA, DA and NE. (I) Schematic illustration depicting the *in vivo* imaging set-up used and perfusion to the brain of flies expressing OA1.0 in the mushroom body (MB, 30y-GAL4-driven). (J) Representative *in vivo* fluorescence images (top left), pseudocolor images (top right), traces (bottom left) and summary (bottom right) of the change in OA1.0 fluorescence measured in the MB horizontal lobe in response to application of DA (500 μM), TA (500 μM), OA (500 μM) and Ep (100 μM). In this and subsequent figures, all summary data are presented as the mean ± SEM, superimposed with individual data. *P < 0.05, ***P < 0.001 and n.s., not significant (for (F)–(H), one-way ANOVA with Tukey's post hoc test; for (J), paired or unpaired Student's *t*-test). Scale bar = 20 μm.

To monitor OA release *in vivo* with high specificity, sensitivity and spatio-temporal resolution, we employed a well-established strategy [42–53] to develop a genetically encoded GPCR activation-based (GRAB) sensor for OA using enhanced green fluorescent protein (EGFP) to report an increase in extracellular OA through an increase in fluorescence intensity. First, we inserted the conformationally sensitive cpEGFP into the third intracellular loop (ICL3) of the β-adrenergic-like OA receptor Octβ2R. Next, we systematically screened the position of the cpEGFP and optimized the linker residues between the GPCR and cpEGFP using site-directed mutagenesis. We then mutated the residues near the ligand binding pocket of Octβ2R to further optimize the performance of the OA sensor. Specifically, we found that introducing at the L7.38 V and I7.41 M substitutions produced an increasing response to OA and we named the GRAB_OA1.0_ (OA1.0) sensor (Fig. 1A and B, and Fig. S1).

When expressed in HEK293T cells, OA1.0 trafficked to the plasma membrane and produced a peak change in fluorescence (ΔF/F_0_) of ∼660% in response to 100 μM of OA (Fig. 1C). To measure the kinetics of the sensor, we used a rapid perfusion system to locally apply OA followed by the OA receptor antagonist epinastine (Ep) and we measured the change in fluorescence using high-speed line scanning. The data were then fitted to obtain an on-rate (τ_on_) and off-rate (τ_off_) of ∼0.02 and ∼1.40 s, respectively (Fig. 1D). We also measured the spectral properties of OA1.0 using both one-photon (1P) and two-photon (2P) excitation, which revealed excitation peaks at ∼500 and ∼920 nm, respectively, and an emission peak at ∼520 nm (Fig. 1E), which were similar to those of other commonly used green fluorescent probes. To confirm that OA1.0 does not activate signaling pathways downstream of Octβ2R (thus not affecting cellular physiology), we measured the β-arrestin and Gs pathway activation using the Tango assay [54]—a cell-based method that quantifies GPCR activation through β-arrestin recruitment and the red cAMP sensor RFlamp, respectively. Cells expressing OA1.0 exhibited negligible β-arrestin-dependent signaling compared with cells expressing WT Octβ2R, even at high concentrations of OA (Fig. 1F, left). Moreover, cells expressing OA1.0 had significantly lower downstream Gs coupling compared with cells expressing WT Octβ2R (Fig. 1F, right).

With respect to its specificity, we found that the OA1.0 signal induced by OA was abolished by Ep and the application of several other neurotransmitters did not produce a detectable change in fluorescence (Fig. 1G, left). Next, we measured the response of OA1.0 to various concentrations of OA, as well as the structurally similar transmitters tyramine (TA), dopamine (DA) and norepinephrine (NE). We found that OA1.0 has an ∼40-fold higher affinity for OA (EC_50_ = ∼200 nM) compared with TA (EC_50_ = ∼8000 nM) and showed a negligible response to DA and NE at all tested concentrations (Fig. 1G, right). However, the utilization of the FSCV method for OA detection does not offer such robust specificity, as we observed significant interference from DA and NE in OA detection despite the relatively minor disruption from TA (Fig. 1H).

To evaluate the specificity of OA1.0 *in vivo*, we generated transgenic flies expressing OA1.0 in the MB (30y-GAL4-driven) and then sequentially applied DA, TA, OA and Ep to the fly brain while performing 2P imaging. We found that neither DA nor TA induced an obvious response, while OA elicited a robust response in OA1.0 fluorescence (with a peak ΔF/F_0_ of ∼100%) that was blocked by Ep (Fig. 1I and J). Together, these data demonstrate that OA1.0 can reliably measure the dynamics of OA release with high specificity for OA.

### OA1.0 can report endogenous OA release signals *in vivo*

**Figure 2.**
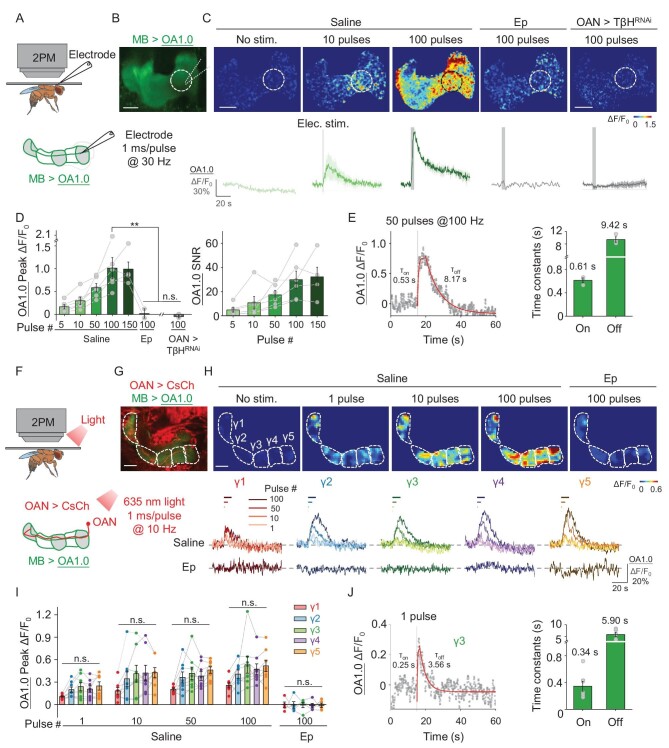
OA1.0 can report the release of OA *in vivo*. (A) Schematic illustration depicting the experimental set-up in which a transgenic fly expressing OA1.0 in the MB (MB247-LexA-driven) is fixed under a two-photon microscope (2PM) and a glass electrode is used to apply electrical stimuli near the MB. (B) Example fluorescence image of OA1.0 expressed in the MB. The dotted circle represents the region of interest used for subsequent analysis. (C) Representative pseudocolor images (top) and corresponding traces (bottom) of the change in OA1.0 fluorescence in response to the indicated number of electrical stimuli in a control fly, a control fly treated with 100 μM of epinastine (Ep) and an OAN (Tdc2-GAL4-driven) > TβH^RNAi^ fly. (D) Summary of peak ΔF/F_0_ (left) and the signal-to-noise ratio (right) measured in response to electrical stimuli for the indicated conditions; *n* = 2–6 flies/group. (E) Left: Time course of ΔF/F_0_ measured in OA1.0-expressing flies in response to 50 electrical stimuli applied at 100 Hz; the rise and decay phases were fitted with a single-exponential function (red traces). Right: Summary of τ_on_ and τ_off_; *n* = 3 flies/group. (F) Schematic illustration depicting the experimental set-up for optogenetic stimulation. (G) Example dual-color fluorescence image of OA1.0 expressed in the MB (green, MB247-LexA-driven) and CsChrimson-mCherry expressed in OANs (red, Tdc2-GAL4-driven). The γ1–γ5 compartments of the MBare indicated using dashed lines. (H) Representative pseudocolor images (top) and corresponding traces (bottom) of the change in OA1.0 fluorescence measured in response to the indicated number of optogenetic stimuli applied either in saline or 100 μM of Ep. (I) Summary of peak ΔF/F_0_ measured in response to optogenetic stimuli; *n* = 8 flies/group. (J) Left: Time course of ΔF/F_0_ measured in the γ3 compartment in response to a single laser pulse; the rise and decay phases were fitted with a single-exponential function (red traces). Right: Summary of τ_on_ and τ_off_; *n* = 7 flies/group. **P < 0.01 and n.s., not significant (for (D), paired or unpaired Student's *t*-test; for (I), one-way ANOVA with Tukey's post hoc test). Scale bar = 20 μm.

To further characterize the release of endogenous OA *in vivo*, we used *Drosophila* expressing OA1.0 in the MB (MB247-LexA-driven), which receives projections from several pairs of OANs, including ventral unpaired median a2 (VUMa2) neurons, ventral paired median 3 (VPM3) neurons, VPM4 neurons, VPM5 neurons and APL neurons [23,55]. To induce the release of endogenous OA in the MB, we applied local electrical stimuli at 30 Hz and observed an incremental increase in fluorescence with an increasing number of stimuli and this response was eliminated by Ep (Fig. 2A-D). Moreover, the response was specific to OA, as no detectable response to electrical stimuli was measured in flies lacking TβH in the OANs (Tdc2-GAL4-driven) (Fig. 2C and D). When we applied 50 electrical stimuli at a frequency of 100 Hz, we measured τ_on_ and τ_off_ rates of ∼0.6 and ∼9.4 s, respectively (Fig. 2E).

To monitor the release of OA in response to the direct activation of OANs *in vivo*, we optogenetically activated OANs (Tdc2-GAL4-driven) in flies expressing CsChrimson-mCherry while simultaneously imaging OA1.0 expressed in the MB (MB247-LexA-driven) (Fig. 2F and G). We found that activating OANs induced a transient increase in OA1.0 fluorescence in the γ1-γ5 compartments of the MB, with the magnitude of the OA1.0 response dependent on the number of light pulses applied; moreover, the peak responses were similar among all five γ compartments (Fig. 2H and I). Importantly, the response for a stimulation of 100 pulses was blocked in all five compartments by Ep, confirming the specificity of the sensor (Fig. 2H and I). We then measured the kinetics of the response using the γ3 compartment as an example and found that a single pulse of a 635-nm laser evoked a measurable increase in OA1.0 fluorescence, with τ_on_ and τ_off_ values of ∼0.34 and ∼5.90 s, respectively (Fig. 2J). Taken together, these results show that OA1.0 can be used *in vivo* to monitor endogenous OA release with high spatio-temporal resolution, high specificity and high sensitivity.

### OA1.0 can detect physiologically evoked OA release in the MB of living flies

**Figure 3.**
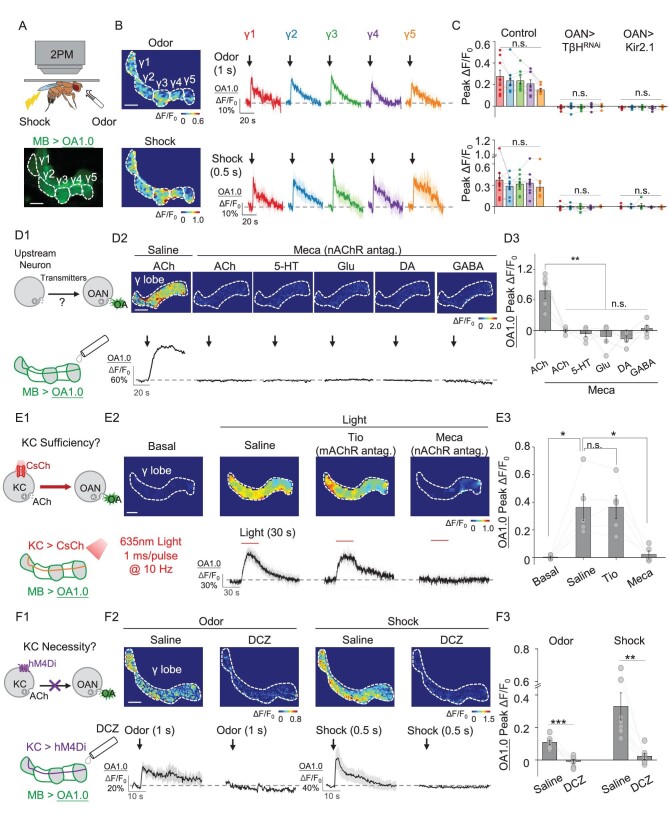
OA1.0 reveals that OA release induced by odor and shock stimuli is activated by ACh released from KCs. (A) Schematic diagram depicting the experimental set-up for 2PM with odor and body shock stimulation in flies expressing OA1.0 in the MB (MB247-LexA-driven), with an example fluorescent image of the MB shown below. (B) and (C) Representative pseudocolor images (B, left), traces (B, right) and summary (C) of the change in OA1.0 fluorescence measured in response to odorant application (top) and body shock (bottom) in OA1.0-expressing flies (*n* = 8–9) and OA1.0-expressing flies co-expressing TβH^RNAi^ (*n* = 6) or Kir2.1 (*n* = 5) in OANs (Tdc2-GAL4-driven). (D) Schematic diagram (D1) depicting the strategy used to apply compounds to the brain of flies expressing OA1.0 in the MB (MB247-LexA-driven). Also shown are representative pseudocolor images (D2, top), traces (D2, bottom) and summary (D3) of the change in OA1.0 fluorescence in response to the indicated compounds (1 mM each) applied in the absence or presence of the nAChR antagonist Meca (100 μM); *n* = 5 flies/group. (E) Schematic diagram (E1) depicting the strategy in which CsChrimson expressed in KCs (R13F02-GAL4-driven) was activated using optogenetic stimulation and OA1.0 fluorescence was measured in the MB (MB247-LexA-driven). Also shown are representative pseudocolor images (E2, top), traces (E2, bottom) and summary (E3) of the change in OA1.0 fluorescence in response to optogenetic stimulation in saline, the muscarinic ACh receptor antagonist Tio (100 μM) and Meca (100 μM); *n* = 5 flies/group. (F) Schematic diagram (F1) depicting the strategy in which hM4Di expressed in KCs (30y-GAL4-driven) was silenced by applying 30 nM of deschloroclozapine (DCZ) and OA1.0 fluorescence was measured in the MB. Also shown are representative pseudocolor images (F2, top), traces (F2, bottom) and summary (F3) of the change in OA1.0 fluorescence in response to odor or electrical body shock in the absence or presence of 30 nM DCZ; *n* = 7 flies/group. *P < 0.05, **P < 0.01, ***P < 0.001 and n.s., not significant (for (C), one-way ANOVA with Tukey's post hoc test; for (D3)–(F3), paired Student's *t*-test). Scale bar = 20 μm.

The conflicting findings regarding the role of OA in aversive olfactory learning [19,28,29] highlight the need to better understand whether OA release can be activated by odor and/or an aversive stimulus such as electric body shock, which can represent either the CS or the US in this type of learning. To address this question, we expressed OA1.0 in the *Drosophila* MB (MB247-LexA-driven) and found that both odorant application and electric body shock induced a time-locked increase in OA1.0 fluorescence in all five γ compartments, with no difference observed among the various compartments (Fig. 3A-C). In contrast, we found no detectable response to either odorant application or electrical shock in flies in which we knocked down TβH expression in OANs or in flies in which OAN activity was suppressed by expressing the inward rectifying potassium channel Kir2.1. As an internal control, direct application of OA still elicited a robust OA1.0 response in both models (Fig. S2).

### OA1.0 reveals that KC activity is both necessary and sufficient for OA release in the *Drosophila* MB

Next, to examine the mechanism underlying OA release in the MB, we attempted to identify the neurons and pathways that regulate OAN activity. Although previous connectomic analyses showed that KCs, the principal neurons in the MB, are the primary cells upstream of OANs (Fig. S3) [56,57], the functional inputs that drive OA release are currently unknown. Given that KCs release the excitatory neurotransmitter acetylcholine (ACh) [58], we perfused ACh onto the γ lobe of the MB and observed an increase in OA1.0 fluorescence that was prevented by the nicotinic ACh receptor (nAChR) antagonist mecamylamine (Meca). Moreover, we found no increase in OA1.0 fluorescence when other neurotransmitters such as 5-hydroxytryptamine (5-HT), glutamate (Glu), DA and γ-aminobutyric acid (GABA) were applied in the presence of Meca (Fig. 3D).

Because the perfusion of exogenous ACh lacks cell-type specificity, we used optogenetics to determine whether selectively activating KCs (R13F02-GAL4-driven) is sufficient to induce OA release in the MB. Consistently with our perfusion experiments, we found that optogenetically activating KCs caused an increase in OA1.0 fluorescence that was blocked by Meca but not the muscarinic ACh receptor antagonist tiotropium (Fig. 3E). Moreover, there is no obvious light-induced OA release in transgenic flies with UAS-CsChrimson but without KC-GAL4 (R13F02-GAL4) (Fig. S4A), ruling out the unspecific effect due to the leaky expression of channelrhodopsin [59]. Together, these results suggest that ACh release from KCs serves as the excitatory signal that drives OA release via nAChRs in the γ lobe of the MB.

To determine whether KCs are required for activating OANs in the MB, we generated transgenic flies expressing both OA1.0 and the inhibitory DREADD (designer receptors exclusively activated by designer drugs) hM4Di [60–62] and found that both odor- and shock-induced OA1.0 signals were abolished when KC activity was suppressed by the hM4Di agonist deschloroclozapine (DCZ) [63] (Fig. 3F). Meanwhile, the DCZ application showed no significant effect on stimuli-induced OA signals in flies without hM4Di (Fig. S4B). Thus, KC activity is both necessary and sufficient for OA release from OANs in the MB.

### OA regulates aversive learning behavior and related synaptic plasticity

**Figure 4.**
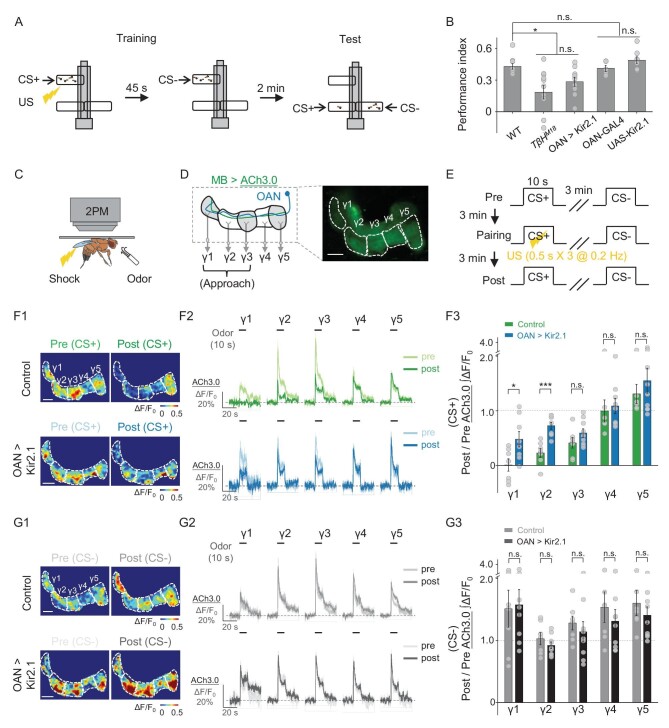
OA plays an essential role in aversive learning and synaptic plasticity in KCs in the MB. (A) Schematic diagram depicting the T-maze protocol for measuring aversive learning in *Drosophila*. (B) Summary of the performance index measured in WT flies and the indicated transgenic flies. OAN-GAL4 and UAS-Kir2.1 served as control groups; *n* = 5–10 for each group. (C)–(E) Schematic diagram (C) depicting the *in vivo* 2PM imaging set-up, a representative fluorescence image (D) and the experimental protocol (E) in which odor-induced changes in ACh3.0 fluorescence (MB247-LexA-driven) in the γ1–γ5 compartments were measured before (pre), during and after (post) pairing. (F) and (G) Representative pseudocolor images (F1, G1) and average traces (F2, G2) of odor-evoked ACh3.0 responses measured in the γ1–γ5 compartments before and after pairing in response to the CS+ odorant (F) and CS– odorant (G) in control flies (top) and OAN-silenced (OAN > Kir2.1) flies (bottom). (F3) and (G3) Summary of the change in odor-evoked ACh release (post/pre responses) after pairing in response to the CS+ odorant (F3) and CS– odorant (G3) in control flies and OAN > Kir2.1 flies; *n* = 6–9 flies/group. *P < 0.05, ***P < 0.001 and n.s., not significant (unpaired Student's *t*-test). Scale bar = 20 μm.

To examine the biological significance of OA release triggered by odorant application and body shock, we measured aversive learning and the coincident time window in flies lacking either OA synthesis or OAN activity. Previous research has demonstrated that the coincidence between the CS and the US is essential for effectively forming associations in aversive learning; furthermore, it has been reported that 5-HT bidirectionally regulates the coincidence time window [64]. We found that both TβH mutant flies and OAN-silenced flies expressing Kir2.1 had significantly reduced learning performance compared with WT flies (Fig. 4A and B). Moreover, unlike flies lacking neuronal tryptophan hydroxylase (Trhn), the rate-limiting enzyme in 5-HT biosynthesis, which have a significantly shortened coincident time window compared with control flies, the coincident time window was unchanged in TβH mutants (Fig. S5). These results suggest that OA plays a key and specific role in aversive learning ability in *Drosophila*.

Given that synaptic plasticity is fundamental to the neuronal basis of learning, the regulation of synaptic plasticity by OAN activity after odor–shock pairing is a potential mechanism underlying the observed aversive learning results. Previous electrophysiological recordings or Ca^2+^ imaging studies in the mushroom body output neuron (MBON) innervating the γ1 compartment (MBON-γ1pedc) suggested that pairing an odorant with dopaminergic reinforcement induces synaptic depression between KCs and the MBON [65–67]. This synaptic depression is correlated with decreased ACh release from KCs [64,68]. Thus, we used the GRAB_ACh3.0_ sensor (ACh3.0) [45] to monitor the ACh release in the γ lobe of the MB (MB247-LexA-driven) (Fig. 4C-E). By comparing the odor-evoked ACh release measured before and after odor–shock pairing in control flies, we observed significant synaptic depression in the γ1, γ2 and γ3 compartments (Fig. S6)—the three compartments known to transmit information to MBONs associated with approach behavior [69]. We then examined the extent of ACh release depression following odor–shock pairing in flies expressing Kir2.1 in the OANs. Our results revealed significant reductions in ACh release depression (i.e. less synaptic depression) in the CS+ response, specifically in the γ1 and γ2 compartments, compared with control flies (Fig. 4F), indicating impaired synaptic plasticity during learning in OAN-silenced flies. In contrast, OAN-silenced flies and control flies showed similar ACh release patterns in response to CS– in all of the γ compartments, indicating that OA is specifically required for learning (Fig. 4G). Taken together, these results suggest that OA plays an essential role in modulating the change in synaptic plasticity induced by odor–shock pairing, thereby amplifying the aversive learning behavior.

### OA regulates aversive learning by modulating US processing via Octβ1R expressed on dopaminergic neurons

**Figure 5.**
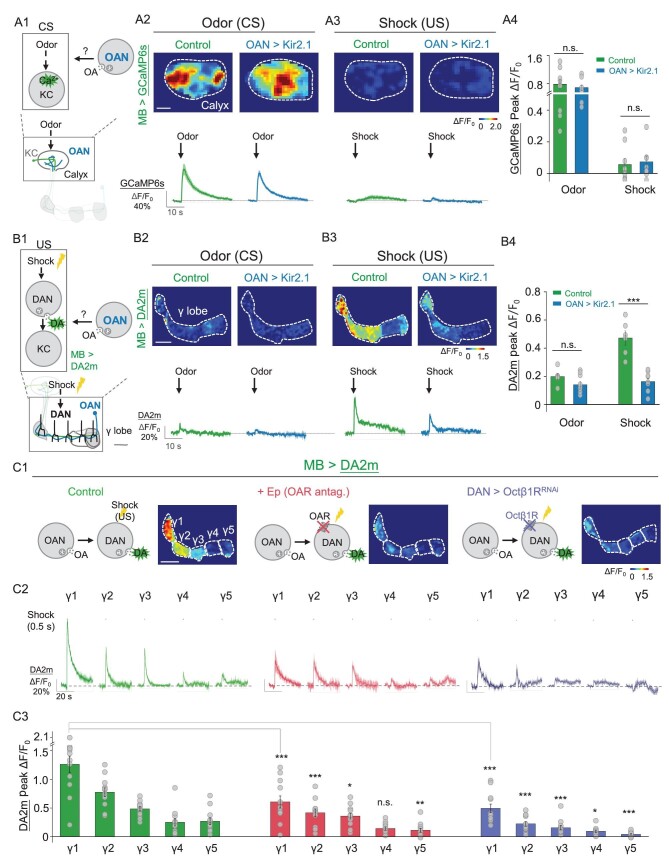
OA is required for driving DA release in response to aversive stimuli. (A) Schematic diagram (A1) showing the strategy for measuring intracellular calcium signals in the MB (MB247-LexA-driven) by expressing GCaMP6s in either control flies or OAN > Kir2.1 flies, in response to theconditioned stimulus (CS) or unconditioned stimulus (US). Also shown are representative pseudocolor images (A2 and A3, top), traces (A2 and A3, bottom) and summary (A4) of calcium signals measured in the calyx in response to odor (A2) or electrical body shock (A3); *n* = 9 flies/group. (B) Schematic diagram (B1) showing the strategy for measuring dopamine (DA) signals in the MB (R13F02-LexA-driven) by expressing the DA2 m sensor in either control flies or OAN > Kir2.1 flies, in response to the CS or US. Also shown are representative pseudocolor images (B2 and B3, top), traces (B2 and B3, bottom) and summary (B4) of DA release measured in the γ lobe in response to odor (B2) or electrical body shock (B3); *n* = 6–9 flies/group. (C) Schematic diagrams (C1) showing DA2 m imaging in flies and representative pseudocolor images whose brain was bathed in saline (left) or saline containing 100 μM of Ep (middle), or DAN > Octβ1R^RNAi^ (TH-GAL4-driven) flies (right) in response to body shock stimuli. Also shown are representative traces (C2) and the summary (C3) of DA release measured in the γ1-γ5 compartments; *n* = 12 flies/group. *P < 0.05, **P < 0.01, ***P < 0.001 and n.s., not significant (unpaired Student's *t*-test). Scale bar = 20 μm.

Synchronization between the CS and the US is required for aversive learning; specifically, information regarding the CS is conveyed by projection neurons to the calyx of the MB for processing by KCs, while information regarding the US is conveyed by dopaminergic neurons (DANs) to the MB lobes for subsequent processing [70]. Consequently, we investigated the specific role of OA in aversive learning. We expressed the calcium sensor GCaMP6s in KCs (MB247-LexA-driven) to measure calcium signals in the calyx, providing information regarding the dynamics of CS processing (Fig. 5A1). The results indicated that OAN-silenced flies exhibited similar KC calcium signals in response to odorant application compared with the control flies (Fig. 5A2 and A4). As anticipated, shock stimuli induced small calcium signals in the KCs of the calyx and no significant differences were observed between OAN-silenced flies and the corresponding control flies (Fig. 5A3 and A4). Additionally, we expressed the GRAB_DA2 m_ (DA2m) sensor [47] in the MB (R13F02-LexA-driven) to measure DA release in the γ lobe, thus capturing the dynamics of US processing (Fig. 5B1). We found that shock-induced DA release in the γ lobe was significantly reduced in OAN-silenced flies (Fig. 5B3 and B4). Moreover, odor stimuli induced small DA transients in the γ lobe and no significant differences were observed between OAN-silenced flies and the corresponding control flies (Fig. 5B2 and B4). Together, these findings suggest that OAN activity modulates US processing, but not CS processing, during aversive learning.

**Figure 6.**
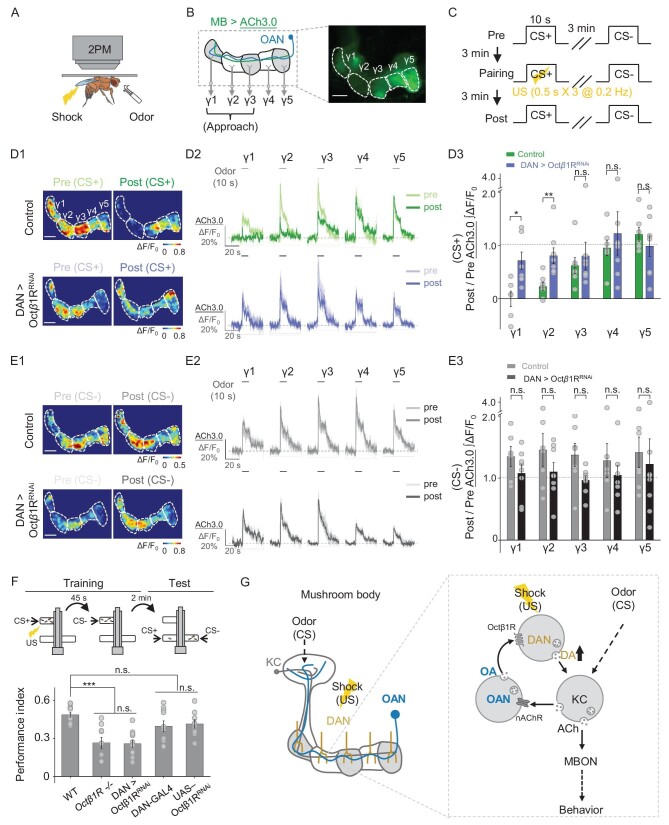
OA acts on DANs via the Octβ1R receptor to modulate aversive learning. (A)–(C) Schematic diagram depicting the *in vivo* 2PM imaging set-up (A), a representative fluorescence image (B) and the experimental protocol (C) in which odor-induced changes in ACh3.0 (MB247-LexA-driven) fluorescence were measured in the γ1–γ5 compartments before, during and after pairing. (D) and (E) Representative pseudocolor images (D1, E1), average traces (D2, E2) and summary (D3, E3) of odor-evoked ACh3.0 responses measured in the γ1–γ5 compartments in response to the CS+ odorant(D) and CS– odorant (E) in the indicated groups; *n* = 6–8 flies/group. (F) Schematic diagram depicting the T-maze protocol (top) and summary of the performance index (bottom) measured in the indicated groups; *n* = 9–12 for each group. (G) Model depicting the proposed mechanism for how OA acts on DANs in the MB to modulate aversive learning. MBON, mushroom body output neuron. *P < 0.05, **P < 0.01, ***P < 0.001 and n.s., not significant (unpaired Student's *t*-test). Scale bar = 20 μm.

To eliminate potential developmental influences on our observations regarding the effect of OA on DA release in response to the US, we applied the OA receptor antagonist Ep to the fly's brain and found that the same individual fly exhibited a significant reduction in shock-induced DA release along the γ lobe when compared before and after the Ep treatment (Fig. 5C, left and middle). Previous studies showed that short-term aversive memory formation requires OA signaling via Octβ1R [25]; we therefore specifically knocked down Octβ1R expression in DANs (TH-GAL4-driven) using RNAi (Fig. 5C, right) to examine whether OA directly affects DA release and found a significant decrease in DA release compared with controls (Fig. 5C, left and right). Based on these results, we then examined whether knocking down Octβ1R expression in DANs affects synaptic plasticity and/or learning. Similarly to our results obtained with OAN-silenced flies (see Fig. 4), we found significant differences in the degree of KC synaptic depression in response to CS + in both the γ1 and γ2 compartments of Octβ1R-knock-down flies compared with control flies. In contrast, we found no significant differences in the γ3, γ4 or γ5 compartments in response to CS+, or in any γ compartment in response to CS– (Fig. 6A-E). To further test the role of Octβ1R expressed in DANs in learning behaviors, we assessed the learning ability of Octβ1R-knockout flies and Octβ1R-knock-down flies at the behavioral level. Our results show that, similarly to synaptic plasticity, both genotypes of flies displayed significantly impaired learning compared with control flies (Fig. 6F). Taken together, these results support a model in which OA boosts aversive learning via Octβ1R in DANs, which enhances the punitive US signals to modulate synaptic plasticity in KCs (Fig. 6G).

## DISCUSSION

Here, we developed a new genetically encoded fluorescent sensor called GRAB_OA1.0_ to detect OA release with high selectivity, sensitivity and spatio-temporal resolution both *in vitro* and *in vivo*. We then used this tool to perform the first detailed study of the spatial and temporal dynamics of OA during aversive learning in *Drosophila*. We found that ACh released from KCs activates OANs, triggering OA release via nAChRs. Notably, we also observed that ACh released from KCs is required for OA release in response to both the CS and the US during aversive learning. Furthermore, by integrating other genetically encoded fluorescent sensors (namely GRAB_DA2 m_ and GRAB_ACh3.0_ to monitor DA and ACh, respectively), we discovered that OA increases shock-induced DA release via Octβ1R, which in turn regulates the corresponding changes in synaptic plasticity in the MB, ultimately facilitating aversive learning.

### Advantages of OA1.0 over other methods for measuring OA

Compared with other methods used to measure OA, OA1.0 offers several advantages. First, OA1.0 exhibits high specificity for OA over most neurotransmitters such as TA, DA and NE. This is particularly important for detecting OA in the presence of other structurally similar molecules, as electrochemical tools such as FSCV cannot distinguish between OA and other chemicals, as shown in here (Fig. 1H) and in previous studies [39–41]. Second, OA1.0 offers sub-second kinetics and is genetically encoded, allowing the non-invasive monitoring of octopaminergic activity *in vivo* with a high recording rate. In contrast, microdialysis has relatively low temporal resolution and requires the placement of a relatively large probe, making it unsuitable for use in small model organisms such as *Drosophila*. Capitalizing on these advantages, we used OA1.0 to monitor OA release *in vivo* in response to a variety of stimuli, gaining new insights into the functional role of OA.

Importantly, OA1.0 can also be expressed in other animal models, including mammals, opening up new opportunities to monitor OA dynamics in a wide range of species. In mammals, OA is classified as a trace amine and exerts its activity through trace amine-associated receptors (TAARs). TAAR1, in particular, has been implicated as a key regulator of monoaminergic and glutamatergic signaling in brain regions relevant to schizophrenia, as demonstrated in knockout and overexpression models in rodents [71,72]. However, studying TAAR1 is challenging due to the presence of various endogenous ligands, including the trace amines β-phenylethylamine (PEA), TA and OA, as well as the monoamine neurotransmitters DA, 5-HT and NE [73]. Thus, the development of robust tools such as OA1.0 that selectively monitor a given trace amine will advance our understanding of specific TAAR-mediated biological effects. Additionally, this strategy can be employed to develop sensors for detecting other key trace amines, providing valuable information regarding the dynamics of these chemicals under both physiological and pathological conditions.

### OA plays a key role in associative learning

OA was initially believed to play a role only in appetitive learning, but not in aversive learning, in invertebrates such as *Drosophila*, honeybees and crickets [19,28,74,75]. However, several studies suggest that OA may indeed be involved in aversive learning, albeit without completely understanding the underlying mechanisms and spatio-temporal dynamics [23,25,29]. Schwaerzel *et al.* first showed that OA has the selective role in *Drosophila*, reporting that TβH mutants had impaired appetitive learning but normal aversive learning [19]. However, it is important to note that the TβH mutants used by Schwaerzel *et al.* were a mixture of homozygous and hemizygous TβH^M18^ flies regardless of sex, as the localization of TβH was to the X chromosome and the homozygous TβH^M18^ females were sterile. Subsequently, Iliadi *et al.* found that both homozygous TβH^M18^ males and females performed impaired aversive conditioning compared with WT flies and heterozygous TβH^M18^ females [29]. Drawing on these previous reports, we used homozygous TβH^M18^ males and females, and obtained results similar to those of Iliadi *et al.*, supporting the notion that OA is required for aversive learning in *Drosophila*.

Moreover, we found that OA release in the γ lobe of the MB plays a crucial role in facilitating the release of DA via Octβ1R, which is selectively coupled to increase intracellular cyclic adenosine monophosphate (AMP) levels by OA [76], in response to shock stimuli. This increased release of DA drives a change in synaptic plasticity between KCs and the MBON and promotes aversive learning [65,67,77–81]. The finding aligns with prior studies showing that DANs are downstream of OANs in reward-based learning [20,21,82], suggesting a conserved role for OA in mediating the ability of DANs to perceive US signals in both positive and negative learning scenarios. It is noteworthy that our study utilized a DA sensor [47] to specifically detect the release of DA itself, providing a more direct assessment of its potential effects on downstream neurons, rather than measuring DAN activity [20,21]. In addition to confirming the involvement of OA in aversive learning, our study also provides novel insights into the underlying input and output circuitry through which OA operates (see Fig. 6G), which potentially indicates that the CS and the US are not entirely independent events within the learning context, but rather one might have an impact on the other.

Nevertheless, further studies are needed to obtain a more comprehensive understanding of the mechanisms through which OA contributes to associative learning. Notably, previous studies found that Octβ1R, expressed in KCs, is involved in aversive learning [25], which operates as a parallel circuit along with the well-known DA–dDA1 (MB-γ)–MBON pathways [83]. Additionally, in the context of appetitive learning, the α1-like OA receptor OAMB has been shown to play a role in engaging octopaminergic signaling in KCs [22]. These intriguing findings suggest that OA may exert a direct effect on KCs to affect associative learning. Thus, further research is needed in order to unravel the complex interactions and mechanisms by which OA modulates associative learning.

### Neuromodulators interact in associative learning

As the primary center of associative memory in *Drosophila*, the MB uses ACh as the predominant excitatory neurotransmitter released from KCs [58]. However, the MB also receives converging inputs from other neuromodulators such as OA, DA, 5-HT and GABA. The interactions between these neuromodulator systems, as well as with ACh, are essential for controlling the states and neuronal computations of the brain [56]. Here, we show that odor- or shock-evoked release of OA requires ACh release from KCs and, in turn, increases DA release, thereby forming a positive feedback loop that is required for learning. However, our imaging results showed that KC activity is both necessary and sufficient for OA release in the γ lobe of the MB, thereby influencing DA release. We did not rule out the possibility that other inputs to OANs, as illustrated in Fig. S3, in which neurons of other classes, aside from KCs, form synaptic connections with OANs, might contribute to DA release. This possibility opens up an intriguing avenue for future research to explore the functional implications of these connections. Additionally, recent research has shown that normal DAN synaptic release during learning requires KC input to DAN [84]. In addition, KCs have been shown to activate GABAergic APL neurons [85] and serotoninergic dorsal paired medial (DPM) neurons [64], both of which provide negative feedback to KCs. GABA release from APL neurons is believed to contribute to odor-specific memory through sparse coding [86], while 5-HT release from DPM neurons regulates the coincidence time window of associative learning [64]. Thus, as the predominant neuron type in the MB, KCs not only associate CS and US signals but also regulate a variety of neuromodulators to form local feedback loops. These local reentrant loops permit moment-by-moment updates of both external (i.e. environmental) and internal information, allowing the appropriate reconfiguration of the flow of information between KCs and MBONs, thus providing behavioral flexibility and the appropriate responses to change the internal and external states of the organism [87].

The interplay between neuromodulators is both complex and essential for shaping the activity of synaptic circuit elements to drive cognitive processes in both invertebrates and mammals. In this respect, our study provides new insights by highlighting the conserved interaction between OA and DA in invertebrates, offering a valuable framework for understanding the complex interplay between DA and other neurotransmitters in associative learning processes. Additionally, a recent study in mammals showed that continuous interactions and updating between ACh and DA signaling in the nucleus accumbens are critical for regulating the striatal output that underlies the acquisition of Pavlovian learning of reward-predicting cues [88,89]. Given the similarities between OA–DA interaction in invertebrates and the ACh–DA interaction in mammals, it is reasonable to speculate that such interactions are a fundamental feature of the central nervous system. The discovery that such conserved interactions exist between distinct neuromodulator systems provides valuable new insights into the mechanisms that underlie cognitive processes and may have important implications with respect to developing new therapies for cognitive disorders.

## METHODS

Detailed methods are provided in the online supplementary data and include the following:

Key resource table

Experimental model and subject details

- Cell lines
- Flies

Detailed methods

- Molecular biology
- Expression of GRAB_OA_ sensors in cultured cells
- Fluorescence imaging of cultured cells
- Tango assay
- RFlamp cAMP measuring assay
- Spectra measurements
- Fast-scan cyclic voltammetry
- Two-photon *in vivo* imaging of flies
- Behavioral assay

Quantification and statistical analysis

- Imaging experiments
- Behavioral experiments
- Statistical analysis

## Supplementary Material

nwae112_Supplemental_Files
