# Supplementary material for: An octopamine-specific GRAB sensor reveals a monoamine relay circuitry that boosts aversive learning
Source: Natl Sci Rev. 2024 Mar 26;11(5):nwae112. doi: 10.1093/nsr/nwae112 (PMC11126161; doi:10.1093/nsr/nwae112)
Supplement: nwae112_Supplemental_Files [file nwae112_supplemental_files.zip › supplementary figure legends.docx]

**Figure S1. Strategy for designing, optimizing, and screening GRAB_OA_ sensors.**

(A) Flowchart depicting the process for developing the OA1.0 sensor with a peak response (ΔF/F_0_) of ~600%.

(B) Amino acid sequence of the OA1.0 sensor, with the various domains and mutated sites indicated. Note that the numbering system corresponds to the start of the IgK leader sequence.

**Figure S2. Changes in OA1.0 fluorescence in response to various stimuli measured in OAN > TβH^RNAi^ and OAN > Kir2.1 flies.**

Representative pseudocolor images (left top), traces (left bottom), and summary (right) of the change in OA1.0 fluorescence measured in response to odor, electrical body shock, and OA perfusion in OAN > TβH^RNAi^ flies (A) and OAN > Kir2.1 flies (B); n = 5-6 flies/group.

Scale bars = 20 μm.

**Figure S3. Summary of the number of synapses between OANs and upstream cells in the MB.** Pedc, peduncle; DPM, dorsal paired medial; APL, anterior paired lateral neuron; MBON, mushroom body output neuron; PPL1, paired posterior lateral 1 cluster neuron; PAM, protocerebral anterior medial cluster neuron; KC, Kenyon cell; TPM, transcripts per million. Version 1.1 of the hemibrain connectome[57] was used for the analysis, and only synapses with a confidence value >0.75 were included.

**Figure S4. KCs release ACh to trigger OA release, related to Fig. 3.**

(A) Light stimulation does not lead to OA release in flies with UAS-CsChrimson but without KC-Gal4 driver, ruling out the unspecific effect caused by leaky expression of channelrhodopsin. Shown are schematics (A1) depicting the in vivo imaging setup in which OA was measured with OA1.0 expressed in KCs (MB247-LexA-driven), while the light pulses (1ms/pulse, 635 nm, 10 Hz) were delivered to the brain of the fly only carrying UAS-CsCh-mCherry, but not KC-GAL4. Also shown are representative pseudocolor images, traces (A2), and summary (A3) of the change in OA1.0 fluorescence in response to light pulses (30 s) in flies without or with KC-GAL4; n = 5 flies/group.

(B) The hM4Di agonist DCZ does not cause significant effect on odor or shock-evoked OA signals in the γ lobe. Shown are schematics depicting the *in vivo* imaging setup in which OA was measured in the γ lobe using OA1.0 expressed in KCs (30y-GAL4-driven) in the absence or presence of 30 nM DCZ (B1). Also shown are representative pseudocolor images (B2, top), traces (B2, bottom), and summary (B3) of the change in OA1.0 fluorescence in response to odor or electrical body shock in the absence or presence of 30 nM DCZ; n = 6 flies/group.

***p < 0.001, and n.s., not significant (unpaired Student’s t-test). Scale bar= 20 μm.

**Figure S5. OA signaling does not regulate the coincidence time window of olfactory learning.**

(A-B) Schematic diagrams depicting odor-shock pairing protocol for measuring how the time interval affects aversive olfactory memory (A) and the effect of varying the inter-stimulus interval (B).

(C-E) Summary of the normalized performance index (Norm. PI) measured with the indicated ISI (C1, D1, E1) and normalized PI-ISI profiles fitted to a sigmoid function, with the corresponding t_50_ values shown (C2, D2, E2). The coincidence time window of olfactory learning is defined as the t_50_ for the sigmoid function and is shown as the shaded area.

Note that the data presented in panels C and D were reproduced and re-plotted from Zeng et al. (2023)[64].

All group data are presented as mean ± SEM.

**Figure S6.** Summary of the relative change in odor-evoked ACh release (post/pre response) following training for the CS+ (left) and CS- (right) measured in wild-type flies.

*p < 0.05, ***p < 0.001, and n.s., not significant (unpaired Student’s t-test)
