# Supplementary material for: An octopamine-specific GRAB sensor reveals a monoamine relay circuitry that boosts aversive learning
Source: Natl Sci Rev. 2024 Mar 26;11(5):nwae112. doi: 10.1093/nsr/nwae112 (PMC11126161; doi:10.1093/nsr/nwae112)
Supplement: nwae112_Supplemental_Files [file nwae112_supplemental_files.zip › Methods.docx]

**KEY RESOURCES TABLE**

| REAGENT or RESOURCE | SOURCE | | IDENTIFIER |
| --- | --- | --- | --- |
| **Chemicals** | | | |
| Octopamine (OA) | Tocris | | Cat #2242 |
| Epinastine (Ep) | Abcam | | Cat #108929-04-0 |
| Acetylcholine (ACh) | Solarbio | | Cat #G8320 |
| 5-hydroxytryptamine (5-HT) | Tocris | | Cat #3547 |
| Histamine (HA) | PerkinElmer | | Cat#NET732 |
| Glutamate (Glu) | Sigma-Aldrich | | Cat #V900408 |
| γ-aminobutyric acid (GABA) | Tocris | | Cat #0344 |
| Tyramine (TA) | Sigma-Aldrich | | Cat #V900670 |
| Norepinephrine (NE) | Sigma-Aldrich | | Cat #A9512 |
| Dopamine (DA) | Sigma-Aldrich | | Cat #H8502 |
| All-*trans*-retinal | Sigma-Aldrich | | Cat #R2500 |
| 3-Octanol (OCT) | Sigma-Aldrich | | Cat #218405 |
| 4-Methylcyclohexanol (MCH) | Sigma-Aldrich | | Cat #153095 |
| Mecamylamine (Meca) | Sigma-Aldrich | | Cat #M9020 |
| Tiotropium bromide (Tio) | Dexinjia Bio & Tech | | N/A |
| Deschloroclozapine (DCZ) | MedChemExpress | | Cat #HY-42110 |
| Mineral oil | Sigma-Aldrich | | Cat #69794 |
| **Cell lines** | | | |
| HEK293T | ATCC | | Cat#CRL-3216;  RRID: CVCL_0063 |
| HTLA cells for Tango Assay | Gift from Bryan L. Roth | | N/A |
| **Recombinant constructs** |  | |  |
| pDisplay-OA1.0-IRES-mCherry-CAAX | This study | | N/A |
| pCMV-OA1.0 | This study | | N/A |
| pCMV- Octβ2R -pHluorin | This study | | N/A |
| pTango-Octβ2R | This study | | N/A |
| pTango-OA1.0 | This study | | N/A |
| pcDNA3.1-CAG-RFlamp | This study | | N/A |
| ***Drosophila* strains** | | | |
| UAS-OA1.0 (chr2) | This study | | N/A |
| LexAop2-OA1.0 (chr2) | This study | | N/A |
| LexAop2-OA1.0 (chr3) | This study | | N/A |
| 30y-GAL4 | Yi Rao, Peking University | | BDSC: 30818 |
| Tdc2-GAL4 | Yi Rao, Peking University | | BDSC: 9313 |
| MB247-LexA | Yi Zhong, Tsinghua University | | N/A |
| R13F02-LexA | Yi Rao, Peking University | | BDSC: 52460 |
| R13F02-GAL4 | Yi Rao, Peking University | | BDSC: 48571 |
| UAS-TβH-RNAi | Jianquan Ni, TsingHua Fly Center | | TH201500898.S |
| UAS-CsChrimson-mCherry | Bloomington *Drosophila* Stock Center | | BDSC: 82180 |
| UAS-CsChrimson-mCherry | Bloomington *Drosophila* Stock Center | | BDSC: 82181 |
| UAS-Kir2.1 | Chuan Zhou, Institute of Zoology, CAS | | N/A |
| UAS-hM4Di | Donggen Luo, Peking University | | N/A |
| Canton-S (W1118) | Yi Rao, Peking University | | N/A |
| Trh01 (Trhn -/-) | Qian et al.[90] | |  |
| TβH mutant (TβH^M18^) | Liming Wang, Shenzhen Bay Laboratory | | BDSC: 93999 |
| LexAop2-ACh3.0 | Jing et al.[45] | | BDSC: 86551 |
| LexAop2-GCaMP6s | Bloomington *Drosophila* Stock Center | | BDSC: 44274 |
| LexAop2-DA2m | Sun et al.[47] | | BDSC: 90880 |
| UAS-Octβ1R-RNAi | Vienna *Drosophila* Resource Center | | VDRC:110537 |
| TH-GAL4 | Yi Rao, Peking University | | N/A |
| **Software** | | | |
| Origin 2019 | OriginLab | http://www.originlab.com/;  RRID:SCR_014212 | |
| ImageJ | NIH | https://imagej.nih.gov/ij/;  RRID: SCR_003070 | |
| Arduino Uno | Arduino.cc | https://www.arduino.cc/en/Guide/ArduinoUno;  RRID:SCR_017284 | |
| MATLAB R2019b | MathWorks | https://www.mathworks.com/;  RRID:SCR_001622 | |

## EXPERIMENTAL MODEL AND SUBJECT DETAILS

**Cell lines**

HEK293T cells were acquired from ATCC and verified by microscopic examination of their morphology and growth curve. The cells were cultured in DMEM (Biological Industries) supplemented with 10% (v/v) fetal bovine serum (FBS, Gibco) and 1% penicillin-streptomycin (Gibco) at 37°C in 5% CO_2_.

**Flies**

In this study, we generated UAS-OA1.0 (attp40), LexAop2-OA1.0 (attp40) and LexAop2-OA1.0 (vk00005) using Gibson assembly to integrate the coding sequence of OA1.0 into the pJFRC28 or modified pJFRC28 vector[91]. The resulting vectors were then injected into *Drosophila* embryos and integrated into attp40 or vk00005 via phiC31 by the Core Facility of Drosophila Resource and Technology, Shanghai Institute of Biochemistry and Cell Biology, Chinese Academy of Sciences.

*Drosophila* were raised at 25°C in 50% humidity and a 12-hour light/dark cycle on a diet of corn meal. For optogenetics experiments, the flies were fed on corn meal containing 400 μM all-trans-retinal immediately after eclosion and kept in total darkness for 8-24 hours prior to imaging experiments.

Detailed fly genotypes used by figures

| Fig. # | Genotype |
| --- | --- |
| Fig. 1 |  |
| 1I and 1J | UAS-OA1.0 / Cyo; 30y-GAL4 / TM2 |
| Fig. 2 |  |
| 2A-2E | LexAop2-OA1.0 / Cyo; MB247-LexA / TM6B |
| 2A-2E | UAS-TβH-RNAi / Tdc2-GAL4; MB247-LexA / LexAop2-OA1.0 |
| 2F-2J | LexAop2-OA1.0 / Tdc2-GAL4; MB247-LexA / UAS-CsChrimson-mCherry |
| Fig. 3 |  |
| 3A-3C | LexAop2-OA1.0 / Cyo; MB247-LexA / TM6B |
| 3C | UAS-TβH-RNAi / Tdc2-GAL4; MB247-LexA / LexAop2- OA1.0 |
| 3C | LexAop2-OA1.0 / Tdc2-GAL4; MB247-LexA / UAS-Kir2.1 |
| 3D | LexAop2-OA1.0 / Cyo; MB247-LexA / TM6B |
| 3E | UAS-CsChrimson-mCherry / LexAop2-OA1.0; R13F02-GAL4 / MB247-LexA |
| 3F | UAS-hM4Di / +; UAS-OA1.0 / +; 30y-GAL4 / + |
| Fig. 4 |  |
| 4A-4B | Canton-S (Control) |
| 4A-4B | TβH^M18^ |
| 4A-4B | Tdc2-GAL4 / UAS-Kir2.1 |
| 4A-4B | Tdc2-GAL4 |
| 4A-4B | UAS-Kir2.1 |
| 4C-4G | LexAop2-ACh3.0 / Cyo; MB247-LexA / TM6B |
| 4C-4G | LexAop2-ACh3.0 / Tdc2-GAL4; MB247-LexA / UAS-Kir2.1 |
| Fig. 5 |  |
| 5A | LexAop2-GCaMP6s / Cyo; MB247-LexA / TM6B |
| 5A | LexAop2-GCaMP6s / Tdc2-GAL4; MB247-LexA / UAS-Kir2.1 |
| 5B | R13F02-LexA / Cyo; LexAop2-DA2m / TM3 |
| 5B | R13F02-LexA / Tdc2-GAL4; LexAop2-DA2m / UAS-Kir2.1 |
| 5C | R13F02-LexA / Cyo; LexAop2-DA2m / TM3 |
| 5C | R13F02-LexA / UAS-Octβ1R-RNAi; LexAop2-DA2m / TH-GAL4 |
| Fig. 6 |  |
| 6A-6E | LexAop2-ACh3.0 / Cyo; MB247-LexA / TM6B |
| 6A-6E | LexAop2-ACh3.0 / UAS-Octβ1R-RNAi; MB247-LexA / TH-GAL4 |
| 6F | Canton-S (Control) |
| 6F | Octβ1R -/- |
| 6F | UAS-Octβ1R-RNAi / +; TH-GAL4 / + |
| 6F | TH-GAL4 |
| 6F | UAS-Octβ1R-RNAi |
| Fig. S2 |  |
| S2A | UAS-TβH-RNAi / Tdc2-GAL4; MB247-LexA / LexAop2-OA1.0 |
| S2B | LexAop2-OA1.0 / Tdc2-GAL4; MB247-LexA / UAS-Kir2.1 |
| Fig. S4 |  |
| S4A | UAS-CsChrimson-mCherry / LexAop2-OA1.0; + / MB247-LexA |
| S4A | UAS-CsChrimson-mCherry / LexAop2-OA1.0; R13F02-GAL4 / MB247-LexA |
| S4B | UAS-OA1.0 / +; 30y-GAL4 / + |
| Fig. S5 |  |
| S4C | Canton-S (Control) |
| S4D | Trhn -/- |
| S4E | TβH^M18^ |
| Fig. S6 |  |
| S6 | LexAop2-ACh3.0 / Cyo; MB247-LexA / TM6B |

**DETAILED METHODS**

**Molecular biology**

Expression clones were generated using the Gibson assembly method. PCR was performed to amplify DNA fragments with ~25-bp overlap using primers (TSINGKE Biological Technology), and T5 exonuclease (New England Biolabs), Phusion DNA polymerase (Thermo Fisher Scientific), and Taq ligase (iCloning) were used to assemble the fragments. Sanger sequencing (TSINGKE Biological Technology) was performed to confirm the plasmid sequences. To characterize the performance of sensors expressed in HEK293T cells, cDNAs encoding the candidate GRAB_OA_ sensors were cloned into the pDisplay vector under the control of the CMV promoter with an upstream IgK leader sequence; a downstream IRES-mCherry-CAAX cassette was included to label the cell membrane and calibrate the sensor’s fluorescence intensity. Spectral properties were measured using plasmids lacking the IRES-mCherry-CAAX cassette. For the Tango assay experiments, genes encoding the WT *Drosophila* Octβ2R and the OA1.0 sensor were cloned into the pTango vector. For the RFlamp cAMP assay, the RFlamp sensor gene was cloned into the pcDNA3.1 vector under the control of the CAG promoter, and OA1.0 and Octβ2R-pHluorin were cloned into the pCMV vector.

**Expression of GRAB_OA_ sensors in cultured cells**

The GRAB_OA_ sensors were screened and characterized in HEK293T cells, which were grown either in 96-well plates or on 12-mm diameter circular coverslips in 24-well plates. At 60-70% confluency, the cells were transfected using polyethyleneimine (PEI) at a PEI:DNA ratio of 3:1, and experiments were conducted 24-36 hours after transfection. For 1P spectra measurements and Tango experiments, the cells were cultured and transfected in 6-well plates; after transfection, the cells were transferred to either a 384-well or 96-well plate for subsequent experiments.

**Fluorescence imaging of cultured cells**

Before imaging, the culture medium was replaced with Tyrode’s solution containing (in mM): 150 NaCl, 4 KCl, 2 MgCl_2_, 2 CaCl_2_, 10 HEPES, and 10 glucose (pH 7.3-7.4). The HEK293T cells grown in 96-well plates were imaged using an Opera Phenix high-content screening system (PerkinElmer), while the cells grown on 12 mm coverslips were imaged using an inverted Ti-E A1 confocal microscope (Nikon). The Opera Phenix high content screening system was equipped with a 20×/0.4- NA objective, a 40×/0.6-NA objective and a 40×/1.15-NA water-immersion objective, a 488-nm laser, and a 561-nm laser; the GRAB_OA_ signal (green fluorescence) was collected using a 525/50-nm emission filter, and the mCherry signal (red fluorescence) was collected using a 600/30-nm emission filter. The fluorescence intensity of the GRAB_OA_ sensor was calibrated using mCherry as the reference. The Nikon confocal microscope was equipped with a 40×/1.35-NA oil-immersion objective and a 488-nm laser; green fluorescence was collected using a 525/50-nm emission filter. To measure the response kinetics, the tip of a glass electrode was placed approximately 10 μm above the cells; this electrode was pulled using a P-97 Flaming/Brown Micropipette Puller (Sutter Instrument) and contained a saturating concentration of agonist or antagonist. A PV800 Pneumatic PicoPump (World Precision Instruments) was used to control the duration of drug delivery. The fast line-scan mode was used to record changes in the local fluorescence signal at the cell membrane, and NIS-Elements software (Nikon) was used to control imaging.

**Tango assay**

HTLA cells were cultured in 6-well plates; at ~70% cell density, the cells were transfected with either wild-type Octβ2R or OA1.0. Twenty-four hours after transfection, the cells were transferred to a 96-well white clear flat-bottom plate, and virous concentrations of OA (ranging from 1 nM to 100 μM) were added to the cells; each concentration was applied in triplicate. The cells were then incubated for ~16 hours, and the bioluminescent signal was measured. To measure the bioluminescent signal, the culture medium was removed, and 40 μl of Bright-Glo substrate (Promega) was added to the wells. The plate was then incubated at room temperature in the dark for 10 minutes, and the bioluminescent signal was measured using a Victor X5 microplate reader (PerkinElmer). Non-transfected cells were used as negative controls.

**RFlamp cAMP measuring assay**

HEK293T cells were transfected with cytoplasmic RFlamp and either the membrane-targeted Octβ2R-pHluorin or OA1.0 sensor. control cells were transfected with only with RFlamp. The cells were then imaged using Operetta CLS (PerkinElmer) before and after the addition of various concentration of OA.

**Spectra measurements**

The 1P spectra of OA1.0 were measured using a Safire 2 microplate reader (Tecan). HEK293T cells were transfected with CMV promoter-driven OA1.0 plasmids (with no other fluorescent proteins); after 24 hours, the cells were dissociated with trypsin and transferred to a clear flat-bottom black-walled 384-well plate for measurement. To detect the excitation spectrum, a gradient of 5-nm (20-nm bandwidth) increments of excitation wavelength was applied from 300–525 nm, and the emission wavelength was fixed at 560 nm (20-nm bandwidth). To detect the emission spectrum, a gradient of 5-nm (20-nm bandwidth) increments of emission wavelength was applied from 495–800 nm, and the excitation wavelength was fixed at 455 nm (20-nm bandwidth). The fluorescence values measured at each wavelength in cells transfected with an empty vector were subtracted as background.

The 2P spectra of OA1.0 were measured using a Bruker Ultima Investigator two-photon microscope equipped with a Spectra-Physics InSight X3 laser. The spectra were measured from 700 nm to 1050 nm at 10-nm increments, and the fluorescence values measured in non-transfected cells were subtracted as background.

**Fast-scan cyclic voltammetry**

Fast-scan cyclic voltammetry (FSCV) was performed using an ElProScan ELP-3 equipped with an EPC10 USB triple potentiostat (HEKA Electronik GmbH, Lambrecht/Pfalz, Germany). A carbon fiber electrode (7-µm diameter, 100-200-µm length, Tokai Carbon Co., Tokai, Japan) was used as the working electrode, and a KCl-saturated Ag/AgCl microelectrode was used as the reference electrode in the two-electrode configuration. All high-speed voltammograms were recorded using a waveform potential from -0.4 V to +1.1 V at a scan rate of 400 V/s with a 200-ms interval. The carbon fiber microelectrode was held at -0.4 V between scans.

**Two-photon *in vivo* imaging of flies**

For the *in vivo* imaging experiments, we used adult female flies within 2 weeks after eclosion. The *in vivo* imaging and stimulation protocols have been adapted from previously established methods[64]. Each fly was mounted onto a customized chamber using tape, and a rectangular section of tape measuring 1 mm x 1 mm above the head was removed. The cuticle between the eyes, air sacs, and fat bodies were carefully removed in sequential order to expose the brain. Throughout the dissection and imaging experiments, the brain was immersed in adult hemolymph-like solution (AHLS) containing (in mM): 108 NaCl, 5 KCl, 5 HEPES, 5 D-trehalose, 5 sucrose, 26 NaHCO_3_, 1 NaH_2_PO_4_, 2 CaCl_2_, and 2 MgCl_2_.

An Olympus FVMPE-RS microscope equipped with a Spectra-Physics InSight X3 dual-output laser was used for the functional imaging experiments. The green fluorescence signals produced by OA1.0, ACh3.0, DA2m, and GCaMP6s were excited using a 920-nm laser and collected through a 495-540-nm filter. The red florescence signals produced by mCherry-tagged CsChrimson were excited using a 1045-nm laser and collected through a 575-630-nm filter.

To apply local electrical stimuli, a glass electrode filling with AHLS (with a tip resistance of 0.2 MΩ) was positioned near the horizontal lobe of the MB, and the stimulation voltage was set to 30–50 V delivered by Grass Instrument S88 Stimulator. For optogenetic stimulation, a 635-nm laser was used to deliver 1-ms pulses at 10 Hz through optical fibers positioned near the fly’s brain. For odor stimulation, the odorant was initially diluted 200-fold in mineral oil, and air was bubbled through the oil at 200 ml/min, combined with pure air delivered at 800 ml/min, and finally delivered to the fly antenna at 1000 ml/min. 3-octanol (OCT) and 4-methylcyclohexanol (MCH) were used for the experiments in Fig. 4 and 6, and OCT was used for the experiments in Fig. 3 and 5. For all odor-shock pairing experiments, either OCT or MCH was randomly assigned as the CS+, with the other odorant serving as the CS-. For body shock stimulation, two copper wires were attached to the fly’s abdomen, with the voltage set to 80 V. The copper wires were adhered to the abdomen of the fruit fly using conductive adhesive, and the resistance measured was 3-10 MΩ. In Fig. 3 and 5, a single shock was delivered with a duration of 0.5 seconds. In Fig. 4 and 6, three shocks were administered with a duration and interval both set at 0.5 seconds. In the experiments shown in Fig. 1I-J and 3D, a small section of the blood-brain-barrier was carefully removed with tweezers before applying the indicated neurotransmitters and compounds.

**Behavioral assay**

These experiments were performed in a dark room at 22°C with humidity ranging from 50-60%. Flies that were 24-72 hours old were selected and transferred to a new tube 12 hours prior to the experiment. Prior to training, the flies were placed in the training arm for 2 min to acclimate.

Each training episode involved ~100 flies, and the odorants OCT and MCH were diluted to 1:67 and 1:100, respectively, in mineral oil. The odorant-containing mineral oil was delivered to the training and testing arms of a T-maze at a rate of 800 ml/min.

During the training phase, the CS+ odorant was introduced to the airflow for 1 min, followed by 12 electric shocks (the US) delivered at 80 V (1.25 s/pulse) via a copper grid located in the training arm; 45 s after the shocks were applied, the CS- odorant was introduced to the airflow for 1 min.

Following training, the flies were transferred to an elevator and given a 2-min acclimation period before testing. During testing, the CS+ and CS- were delivered from the two ends of the arms for a duration of 2 min, during which the flies were allowed to make their choice. The number of flies in each arm (N) was recorded after testing, and the performance index (PI) of each trial was calculated.

An Arduino microcontroller board was used to synchronize the delivery of various stimuli, including odorants, shock, and 635-nm light.

## QUANTIFICATION AND STATISTICAL ANALYSIS

**Imaging experiments**

Images were processed using ImageJ software (National Institutes of Health). The change in fluorescence (ΔF/F_0_) was calculated using the formula [(F-F_0_)/F_0_], where F_0_ is the baseline fluorescence with subtraction of background. Specifically, the value of F0 was determined by subtracting the intrinsic fluorescence of the probe from the instrumentally provided spontaneous fluorescence. The signal-to-noise ratio (SNR) was calculated by dividing the peak response by the standard deviation of the baseline fluorescence. The area under the curve was determined using the integral of the fluorescence response (∫ΔF/F_0_).

**Behavioral experiments**

The performance index (PI) was calculated using the formula [(N_CS-_ – N_CS+_) / (N_CS+_ + N_CS-_)]. To minimize the potential influence of innate bias, each PI data point was derived using the average of two trials; in one trial OCT served as the CS+, and in the other trial MCH served as the CS+. This average was then calculated using the formula [(PI_OCT CS+_ + PI_MCH CS+_) / 2].

**Statistical analysis**

Origin 2019 (OriginLab) was used to perform the statistical analyses. Unless otherwise specified, all summary data are presented as the mean ± sem. The paired or unpaired Student’s *t*-test was used to compare two groups, and a one-way analysis of variance (ANOVA) with Tukey’s post hoc test was used to compare more than two groups. All statistical tests were two-tailed, and differences were considered statistically significant at *P* < 0.05. **P* < 0.05; ***P* < 0.01; ****P* < 0.001; and n.s., not significant.

## REFERENCES

90. Qian, Y., et al., *Sleep homeostasis regulated by 5HT2b receptor in a small subset of neurons in the dorsal fan-shaped body of drosophila.* eLife, 2017. **6**.

91. Pfeiffer, B.D., J.W. Truman, and G.M. Rubin, *Using translational enhancers to increase transgene expression in Drosophila.* Proc Natl Acad Sci U S A, 2012. **109**(17): p. 6626-31.
