# Supplementary figures and images for: An octopamine-specific GRAB sensor reveals a monoamine relay circuitry that boosts aversive learning
Source: Natl Sci Rev. 2024 Mar 26;11(5):nwae112. doi: 10.1093/nsr/nwae112 (PMC11126161; doi:10.1093/nsr/nwae112)

Figure S1

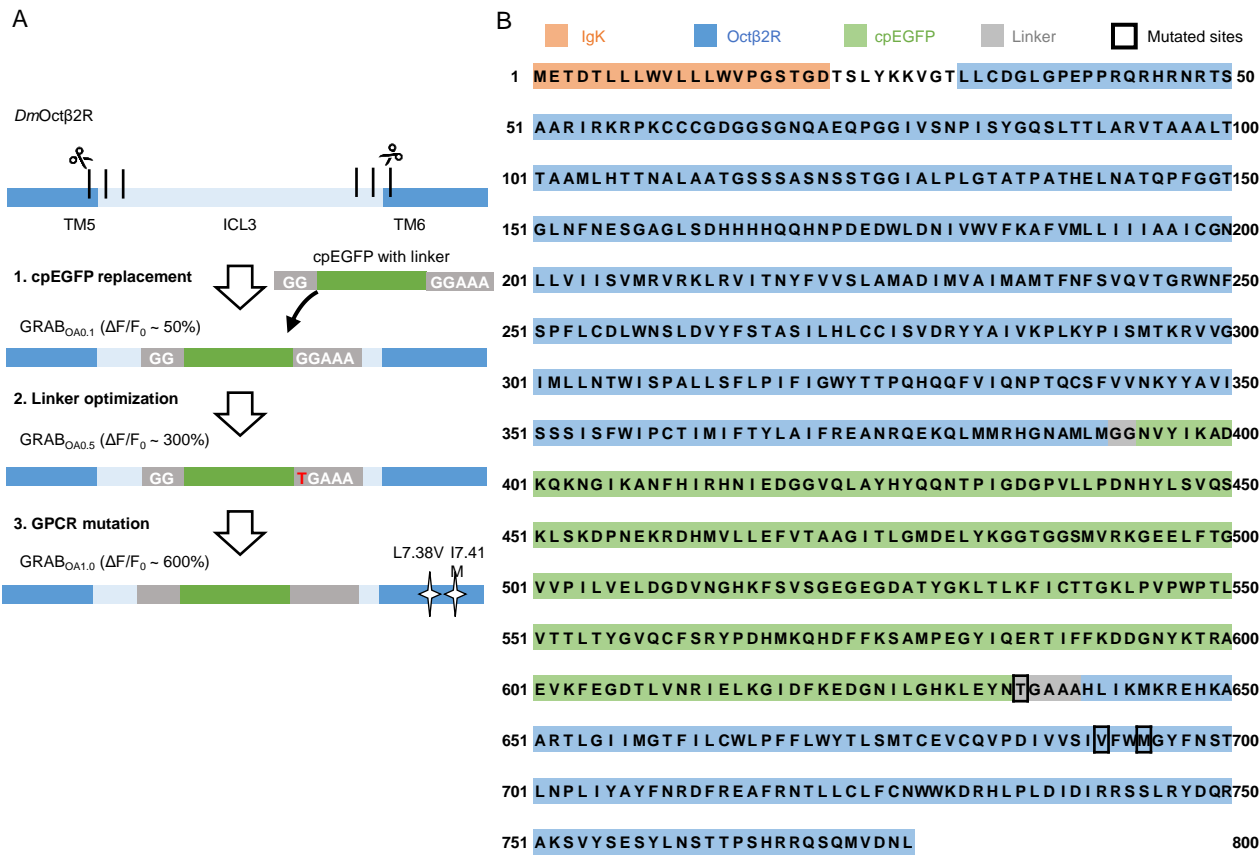

Figure S2

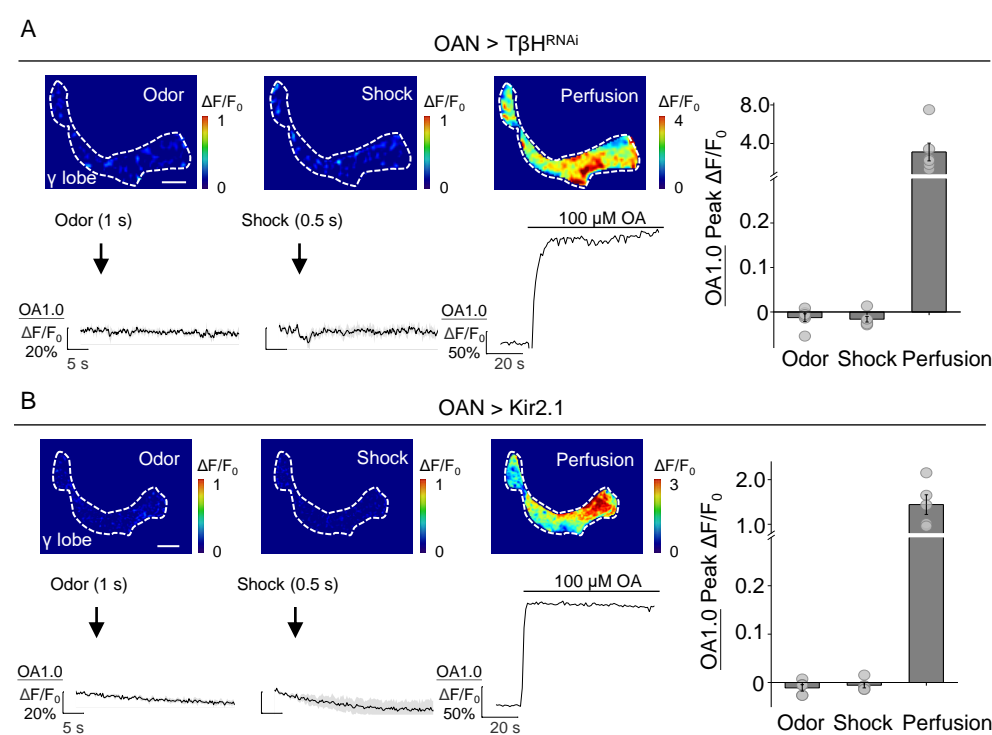

Figure S3

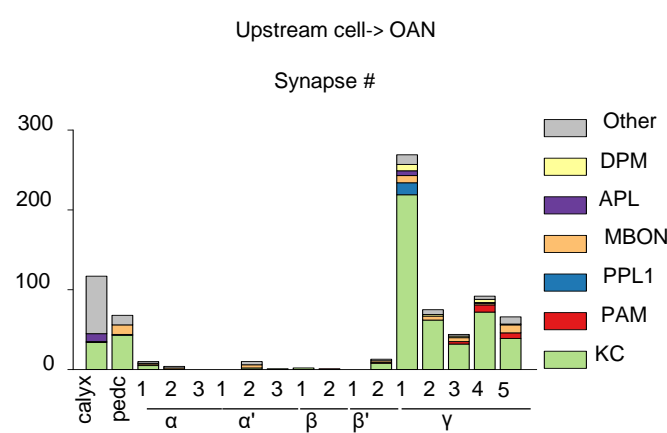

Figure S4

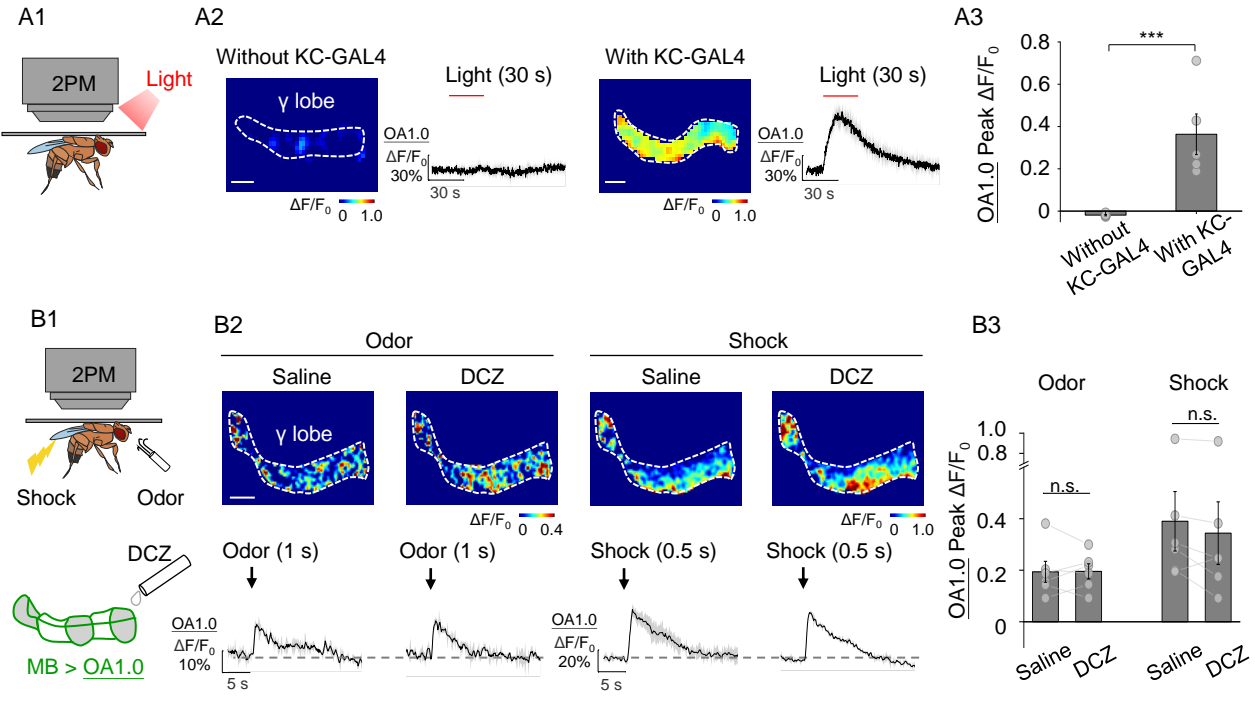

Figure S5

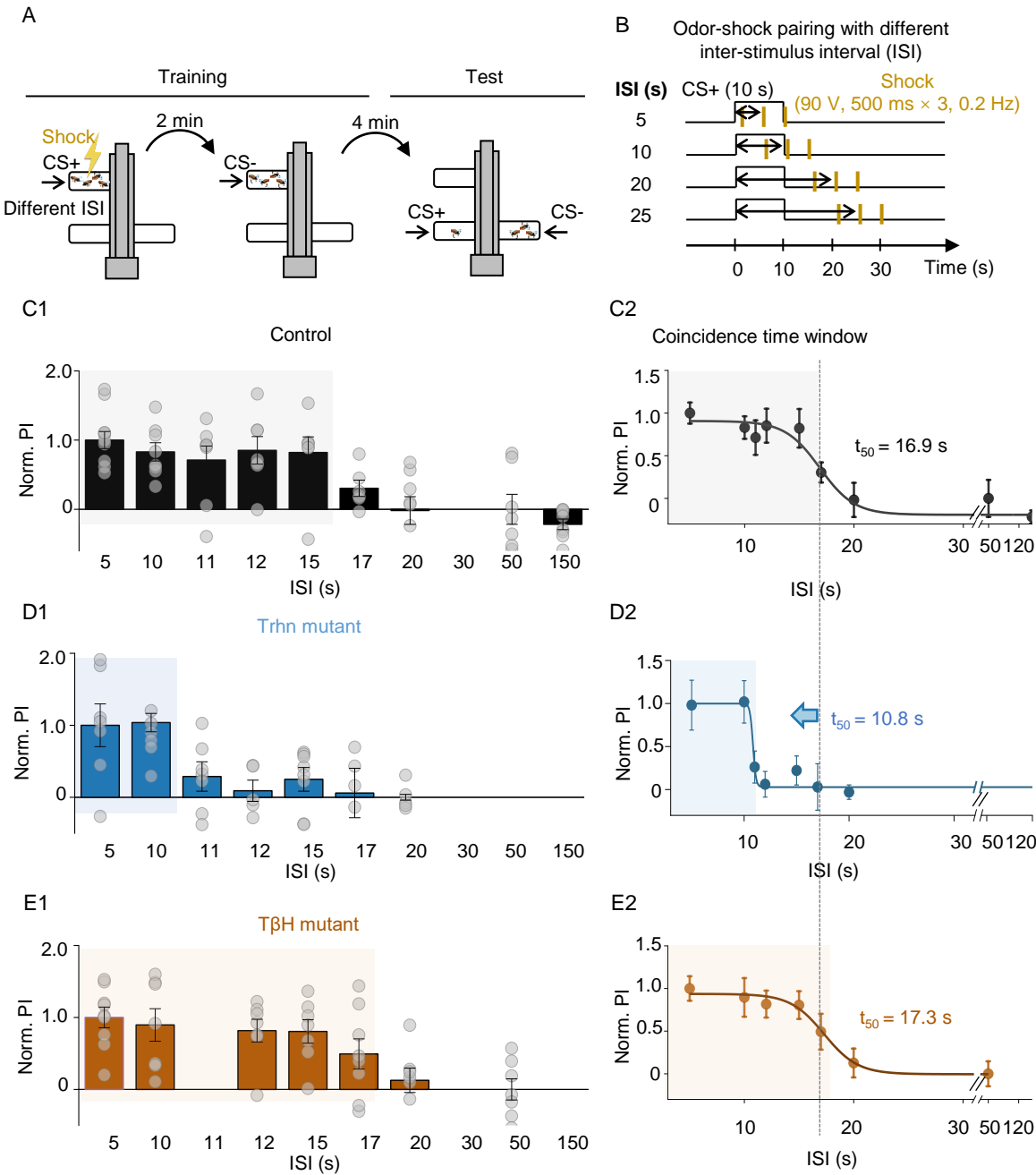

Figure S6

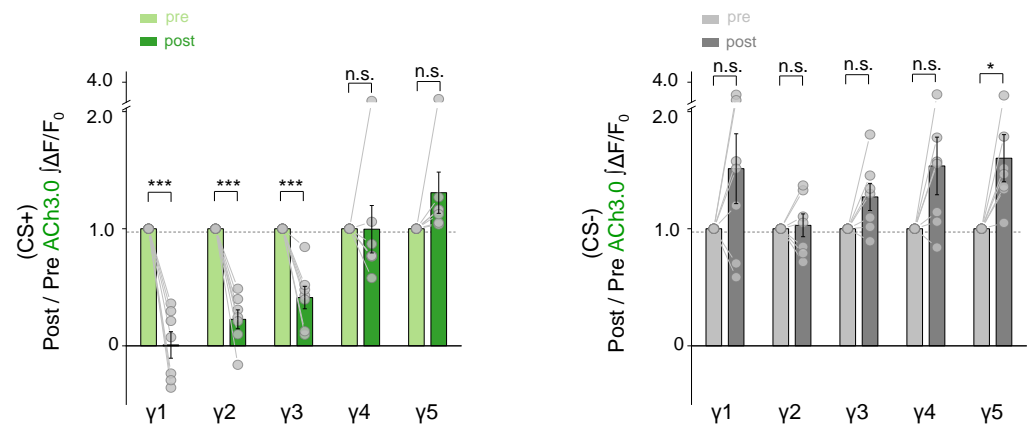

Supplement: nwae112_Supplemental_Files [file nwae112_supplemental_files.zip › supplementary figures.pdf]
